# Predictors of Health-Related Quality of Life in Outpatients with Cirrhosis: Results from a Prospective Cohort

**DOI:** 10.1155/2013/479639

**Published:** 2013-12-22

**Authors:** Maja Thiele, Gro Askgaard, Hans B. Timm, Ole Hamberg, Lise L. Gluud

**Affiliations:** ^1^Department of Medicine, Copenhagen University Hospital Koge, 4600 Koege, Denmark; ^2^Department of Medicine, Copenhagen University Hospital Gentofte, 2900 Hellerup, Denmark; ^3^Department of Gastroenterology and Hepatology, Odense University Hospital, 5000 Odense, Denmark; ^4^Department of Hepatology, Copenhagen University Hospital Rigshospitalet, 2100 Copenhagen, Denmark; ^5^Department of Medicine, Copenhagen University Hospital Glostrup, 2600 Glostrup, Denmark; ^6^Gastrounit, Copenhagen University Hospital Hvidovre, 2650 Hvidovre, Denmark

## Abstract

*Background.* Cirrhosis may lead to a poor health-related quality of life (HRQOL), which should be taken into consideration when addressing the cirrhotic outpatient. *Methods.* Prospective cohort study evaluating predictors of HRQOL in outpatients with cirrhosis. Patients with overt hepatic encephalopathy at baseline were excluded. HRQOL was evaluated at baseline using the six point Chronic Liver Disease Questionnaire. Predictors of low quality of life scores (<4 points) and mortality were analyzed using multivariable logistic regression. *Results.* In total, 92 patients were included (mean age 61 years, 59% male). Nineteen patients died (mean duration of follow-up 20 months). The mean Child-Pugh score was 6.9. Twenty percent had a poor HRQOL judged by the Chronic Liver Disease Questionnaire score and 45% had covert hepatic encephalopathy. The only predictors of poor HRQOL were the Child-Pugh score (*β*=0.45;*P* = 0.013), nonalcoholic etiology of cirrhosis (*β*=−2.34;*P* = 0.009), and body mass index (*β*=−0.20;*P* = 0.023). The body mass index predicted poor HRQOL independently of the presence of ascites and albumin level. *Conclusions.* The body mass index was associated with a low HRQOL. This suggests that malnutrition may be an important target in the management of patients with cirrhosis.

## 1. Introduction

The prognosis of cirrhosis has improved following the development of a number of effective interventions [1–3]. The improvements include the management of gastrointestinal bleeding, hepatorenal syndrome, spontaneous bacterial peritonitis, hepatocellular carcinoma, and hepatic encephalopathy (HE) [4–8]. The health-related quality of life (HRQOL) is therefore becoming increasingly important [9–13]. Most quality of life studies have used generic questionnaires, which allow for comparisons between different groups of patients. These questionnaires will provide an overall picture of the wellbeing of participants. Patients with cirrhosis have specific somatic and cognitive symptoms that may affect their HRQOL [12, 14, 15]. These symptoms may not be captured by generic scales [9, 13, 16, 17]. Questionnaires specifically for patients with chronic liver disease have therefore been developed [14, 18].

Identifying factors associated with HRQOL may help improve patient care and guide future research [12]. This is especially the case for long-term care in an outpatient setting. We therefore performed a prospective cohort study aimed at investigating the prognosis and predictors of HRQOL in patients with cirrhosis followed up at an outpatient setting.

## 2. Materials and Methods

### 2.1. Included Subjects

From February 2008 to May 2012 we conducted a prospective cohort study on patients with cirrhosis recruited from two Danish liver outpatient clinics. Patients were eligible for inclusion if they had histological or clinical cirrhosis and were able to read Danish. Patients with overt HE (West Haven Criteria Grades 2 to 4) and concurrent malignancy were excluded. The study protocol conformed to the ethical guidelines of the 1975 Declaration of Helsinki and was approved by the Danish ethics committee.

Patients were identified at their regular visits to the participating clinics or through the Danish case-mix system of diagnostic codes (dkDRG; Diagnosis Related Groups, based on the ICD-10 classification system). Patients identified electronically were invited to participate by telephone. Patients who agreed to participate completed a written informed consent at the inclusion visit.

At inclusion, demographic data, the patient history, and medication were recorded. A full physical examination and a broad screening panel of tests (including liver function tests, haematology, creatinine, and electrolytes) were performed. The Child-Pugh score was calculated. Evidence of covert HE [17] was evaluated clinically and using the continuous reaction time test (CRT), which is a validated computerized psychometric test [19–24]. The test records reaction times to sound stimuli. Test results are expressed as reaction time percentiles (10, 50, and 90). An index value for the reaction time variance (index value = 50 percentile/90 percentile − 10 percentile) is calculated. Lower index values indicate higher reaction time variance with values below 1.900 suggesting HE in patients with cirrhosis.

HRQOL was evaluated using the six-point, 29-item Chronic Liver Disease Questionnaire (CLDQ) [18]. The questionnaire covers six domains: abdominal symptoms, fatigue, worry, activity, and systemic symptoms. The questionnaire was validated in a pilot study cohort of 15 patients (after backward and forward translation into Danish).

### 2.2. Statistical Analyses

Patient characteristics were summarized as proportions with means and standard deviations/range. The CLDQ score was classed as low (<4) or high (4–6). Predictors of CLDQ scores and mortality were analyzed using binary logistic regression. Multivariable analyses were performed using backward elimination. The predictors were age, gender, body mass index (BMI), etiology of liver cirrhosis, employment, marital status, comorbidities, previous hepatic decompensation, covert hepatic encephalopathy, Child-Pugh score, ascites, ongoing alcohol abuse, albumin, and hyponatremia. Statistical analyses were performed using STATA version 12 (Stata Corp., College Station, Texas, US).

## 3. Results

### 3.1. Patient Characteristics

A total of 92 patients were included and followed for a mean duration of 20 months (range 3 to 52 months). Four patients withdrew their consent regarding the CRT test and the HRQOL questionnaire. Five patients did not complete the CRT test or the HRQOL scores because they had to be hospitalized due to worsening of their underlying liver disease. All 92 patients continued in the follow-up cohort and were included in the outcome analysis.

The mean age of included patients was 61 (SD 8.7; range 41 to 83 years) and 54 (59%) were men. The mean body mass index was 24 (SD 4.0; range 16 to 35). Most patients had cirrhosis due to alcoholic liver disease (Table 1). The remaining patients had autoimmune liver diseases (4), nonalcoholic steatohepatitis (2), chronic hepatitis C infection (3), hemochromatosis (1), or cryptogenic cirrhosis (3).

Fifty-two percent of the patients were classed as Child-Pugh class A. The mean Child-Pugh score was 6.9 (SD 1.7; range 5 to 12). Seventy-seven patients had clinical signs of decompensation prior to inclusion. At baseline, 26 patients (28%) had ascites and 41 patients (45%) were diagnosed as having covert HE. Sixty-one patients were treated with lactulose, 61 with beta-blockers, 57 with loop diuretics, and 35 with spironolactone. Eighty-three received vitamins.

During followup, 19 patients died (21%), including seven classed as Child-Pugh class A at baseline (Table 2). Most patients died from liver related causes. Even though patients with concurrent malignancy were excluded, six fatalities were due to cancer disease. Four of these were hepatocellular carcinoma, stressing the importance of this cancer even in a population consisting largely of patients with alcoholic cirrhosis. One patient underwent successful liver transplantation and two patients received a transjugular intrahepatic portosystemic shunt. Spontaneous bacterial peritonitis was diagnosed in six patients and 34 developed other bacterial infections that required hospitalization. Seventeen patients were admitted with upper gastrointestinal bleeding, 25 developed overt HE, and six developed hepatorenal syndrome. Seven patients were admitted with an ischemic stroke (Table 3).

In univariable logistic regression, the Child-Pugh score and albumin predicted mortality (regression coefficient (*β*) 0.362; *P* = 0.018 and *β* = −0.129; *P* = 0.012). None of the remaining variables were associated with mortality (Table 4). In multivariable regression analysis, the Child-Pugh score remained the only predictor of mortality after backward elimination.

**Figure 1:**
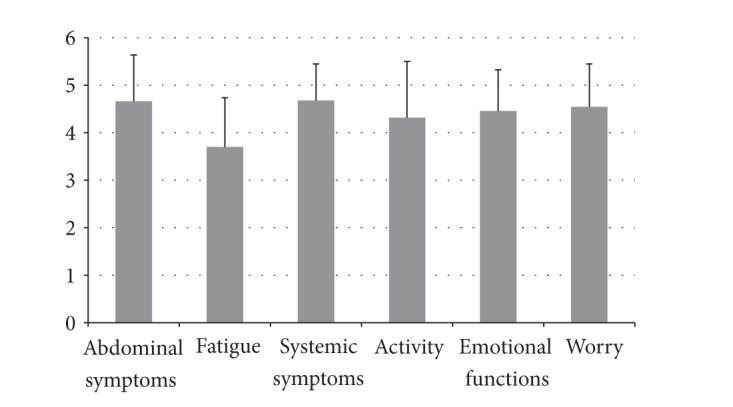
Means with standard deviations for the Chronic Liver Disease Questionnaire domains.

The mean CLDQ score was 4.4 (SD 0.7; range: 2.8 to 5.9). Eighteen patients (20%) had low CLDQ scores. The most frequent complaint was fatigue (mean score 3.7; SD 1.0; range 1 to 6 points) and most commonly patients had trouble lifting or carrying heavy objects (Figure 1). Univariable analysis found that patients with a low CDLQ score were more likely to have a high Child-Pugh score (*β* = 0.454; *P* = 0.013), other causes of cirrhosis than alcohol (*β* = −2.343; *P* = 0.009), and a low BMI (*β* = −0.202; *P* = 0.023). The association with BMI was independent of the presence of ascites, low albumin or the Child-Pugh score. None of the remaining variables were associated with low CLDQ scores (Table 4). In multivariable analysis, the Child-Pugh score and etiology other than alcohol were independent predictors of CLDQ scores (*β* = 0.854; *P* = 0.006 and *β* = −2.583; *P* = 0.039) but not BMI (*β* = −0.210; *P* = 0.060).

## 4. Discussion

This prospective cohort study showed that one in five outpatients with cirrhosis had a low health-related quality of life. The finding was surprising considering the high proportion of patients without signs of hepatic decompensation. The severity of the underlying liver disease predicted both poor HRQOL and mortality. The nutritional status estimated by the BMI was a predictor of HRQOL, independent of the presence of ascites or low albumin. Patients with a low BMI were more likely to have a low HRQOL.

The relatively small sample size is the main limitation of the present study and may explain why the Child-Pugh score, non-alcoholic etiology, and BMI were the only predictors of quality of life. However, our results concur with studies showing that the Child-Pugh score is associated with the quality of life. Our results concur with studies showing that the Child-Pugh score is associated with the quality of life [12, 14]. The association between a low BMI and a low quality of life has been identified for patients with other disease categories but not for patients with cirrhosis [25, 26]. The association between a low BMI and a low quality of life score was independent of the severity of the underlying liver disease, presence of ascites, and albumin level. This suggests that interventions aiming at an improved overall nutritional status of cirrhotic patients may improve their quality of life. We recommend following the International Society for Hepatic Encephalopathy and Nitrogen Metabolism consensus statement regarding nutritional management of hepatic encephalopathy in patients with cirrhosis [27]. Frequent small meals rich in vegetables and dairy protein supplemented with a night time snack of complex carbohydrates may be of benefit to all cirrhotics with a BMI below 25. Accordingly, studies on patients with cirrhosis have found a beneficial effect on the HRQOL of branched chain amino acids and late evening nutritional supplements, which resulted in minor weight gains [28–30].

Our results do not allow for a recommendation of the optimal BMI in chronic liver disease, neither does our study offer a causal explanation of the association between lower BMI and low HRQOL. A low BMI may cause low HRQOL, for example, due to decreased resilience. Alternatively, the body weight of patients with a low HRQOL may decrease, for example, due to inadequate food intake. However, as excess body weight has been associated with increased risk of HCC in chronic liver disease and disease progression in alcoholic liver disease, it can be speculated that BMI in patients with cirrhosis should not exceed 25 [31, 32]. Likewise, avoiding obesity associated with coronary heart disease and diabetes is of great importance, especially as the incidence of NASH cirrhosis is increasing.

The CLDQ is developed and validated in cohorts of patients with all types of liver diseases [18]. We did however find that patients with nonalcoholic etiologies of liver disease had significantly lower HRQOL than patients with alcoholic cirrhosis. The reason for this is unclear and opposes prior findings [14].

In agreement with previous studies, 60% of included patients had covert HE [33, 34]. We found no association between CLDQ scores and HE as judged by the continuous reaction times. Unlike studies on overt HE [9, 13, 16, 35], the evidence on the impact of covert HE on HRQOL is less conclusive [9, 13, 36–39]. However, we cannot exclude that our study would have generated different results if the sample size was larger.

Although most of the included patients had compensated liver disease at baseline, the prognosis was severe. Twenty-one percent of included patients died. Seven of nineteen deaths occurred in patients who were classed as Child-Pugh group A. Most of these patients had previous signs of decompensated liver disease with ascites, variceal bleeding, spontaneous bacterial peritonitis, or overt HE. Our results support the theory that decompensating events as well as Child-Pugh scores predict long-term prognosis in cirrhosis [3]. The increased risk of infections concurs with previous evidence [40, 41]. The relatively high number of strokes may reflect the coagulopathy that is seen in chronic liver disease [42].

In conclusion, this study found that the prognosis of patients with cirrhosis is severe. The finding suggests that these patients should be followed at outpatient clinics as even patients with a low Child-Pugh class may benefit from regular visits. Additional studies are needed to identify the most efficient management strategies in order to improve the prognosis as well as the quality of life. Interventions directed against malnutrition may help achieve this goal.

## Figures and Tables

**Table 1:** Patient characteristics at inclusion.

Variable	Mean ± SD (range) *N* (%)
Followup (months)	19.9 ± 16.0 (3–52)
Male gender	54 (59)
Employed	14 (15)
Married or similar	51 (55)
Age (years)	61.5 ± 8.7 (41–83)
Alcoholic liver cirrhosis	79 (86)
Child-Pugh score	6.8 ± 1.6 (5–12)
Child-Pugh class A/B/C	46/38/8
International normalized ratio	1.28 ± 0.3 (0.9–3.1)
ALT (international units)	32 ± 25 (3–163)
Creatinine (*μ*mol/L)	86 ± 35 (43–221)
Sodium (mmol/L)	137 ± 6 (101–146)
Ammonia (*μ*mol/L)	37 ± 34 (0–91)
Albumin (g/L)	39 ± 6 (27–50)
Ongoing alcohol abuse	39 (42)
Ascites present	26 (28)
Prior decompensation	
Ascites	59 (64)
Hepatic encephalopathy	21 (23)
Varices	41 (45)
Prior variceal bleeding	13 (14)
Comorbidities	
Lung disease	11 (12)
Heart disease*	30 (33)
Kidney disease	12 (13)
Diabetes	18 (20)
Prior malignancy	8 (9)

*includes arterial hypertension and atrial fibrillation. ALT: alanine

aminotransferase.

**Table 2:** Causes of death during followup.

Causes	Child-Pugh class
Gastrointestinal bleeding (1), variceal bleeding (2), hepatorenal syndrome (1), metastatic oropharynx cancer (1), and HCC (2).	A
Metastatic lung cancer (2), progressive liver failure (2), HCC (1), sepsis (1), and unknown (1).	B
Gastrointestinal bleeding (1), progressive liver failure (2), HRS (1), and HCC (1).	C

HCC: hepatocellular carcinoma; HRS: hepatorenal syndrome.

**Table 3:** Clinical outcomes during followup.

Event	*N* (%)
Death	19 (21)
Transplantation	1 (1)
TIPS	2 (2)
Hepatic encephalopathy	25 (27)
Hepatorenal syndrome	6 (7)
Nonvariceal gastrointestinal bleeding	17 (18)
Variceal bleeding	5 (5)
Hepatocellular carcinoma	4 (4)
Spontaneous bacterial peritonitis	6
Bacterial infections	34
Other events, requiring hospitalisation	25

TIPS: transjugular intrahepatic portosystemic shunt.

**Table 4:** Univariable regression analysis of potential predictors for mortality and health-related quality of life.

Variable	Mortality		CLDQ score < 4	
	Regression coefficient β	*P* value	Regression coefficient β	*P* value
Age	0.017	0.576	−0.020	0.507
Gender	−0.580	0.264	−0.945	0.091
Body mass index	−0.147	0.083	**−0.202**	**0.023**
Employment	−0.405	0.619	−0.539	0.519
Marital status	0.095	0.855	−0.841	0.133
Nonalcoholic etiology of cirrhosis	−0.891	0.196	**−2.343**	**0.009**
Comorbidities				
Heart disease	0.598	0.267	0.270	0.634
Pulmonary disease	—	—	−0.219	0.798
Renal disease	0.758	0.263	−0.515	0.537
Diabetes	0.557	0.360	−0.644	0.437
Previous malignancy	0.921	0.238	−0.348	0.763
Previous hepatic decompensation				
Ascites	0.459	0.425	−0.270	0.634
Hepatic encephalopathy	0.642	0.267	−0.707	0.315
Esophageal varices	0.118	0.820	−0.879	0.140
Child-Pugh score	**0.362**	**0.018**	**0.454**	**0.013**
Ascites at inclusion	−0.142	0.807	0.752	0.199
Minimal hepatic encephalopathy	−0.013	0.982	0.160	0.778
Hyponatremia	−0.002	0.679	−0.003	0.521
Albumin	**−0.129**	**0.012**	−0.083	0.109
Ongoing alcohol abuse	−0.064	0.903	−0.491	0.379

CLDQ: Chronic Liver Disease Questionnaire.

Results in bold refers to statistically significant predictors of mortality and CLDQ.

## Abbreviations

BMI: Body mass index
CLDQ: Chronic Liver Disease Questionnaire
CRT: Continuous reaction time test
HE: Hepatic encephalopathy
HRQOL: Health-related quality of life.
